# Supplementary material for: Iterative improvement in the automatic modular design of robot swarms
Source: PeerJ Comput Sci. 2020 Dec 7;6:e322. doi: 10.7717/peerj-cs.322 (PMC7924708; doi:10.7717/peerj-cs.322)
Supplement: Supplemental Information 3 [file peerj-cs-06-322-s003.zip › argos3/doc/api/standalone/a00496_source.html]

ARGoS: plugins/robots/generic/control\_interface/ci\_gripper\_actuator.cpp Source File


- Main Page
- Related Pages
- Namespaces
- Classes
- Files

- File List
- File Members

# plugins/robots/generic/control\_interface/ci\_gripper\_actuator.cpp

Go to the documentation of this file.

```
00001 
00007 #include "ci_gripper_actuator.h"
00008 #include <argos3/core/utility/math/range.h>
00009 
00010 #ifdef ARGOS_WITH_LUA
00011 #include <argos3/core/wrappers/lua/lua_utility.h>
00012 #endif
00013 
00014 namespace argos {
00015 
00016    /****************************************/
00017    /****************************************/
00018 
00019    CRange<Real> UNIT(0.0f, 1.0f);
00020 
00021    /****************************************/
00022    /****************************************/
00023 
00024 #ifdef ARGOS_WITH_LUA
00025    /*
00026     * The stack must have no values
00027     */
00028    int LuaGripperLock(lua_State* pt_lua_state) {
00029       /* Get wheel speed from stack */
00030       if(lua_gettop(pt_lua_state) != 0) {
00031          return luaL_error(pt_lua_state, "robot.gripper.lock() expects no arguments");
00032       }
00033       /* Perform action */
00034       CLuaUtility::GetDeviceInstance<CCI_GripperActuator>(pt_lua_state, "gripper")->Lock();
00035       return 0;
00036    }
00037 
00038    /*
00039     * The stack must have no values
00040     */
00041    int LuaGripperUnlock(lua_State* pt_lua_state) {
00042       /* Get wheel speed from stack */
00043       if(lua_gettop(pt_lua_state) != 0) {
00044          return luaL_error(pt_lua_state, "robot.gripper.unlock() expects no arguments");
00045       }
00046       /* Perform action */
00047       CLuaUtility::GetDeviceInstance<CCI_GripperActuator>(pt_lua_state, "gripper")->Unlock();
00048       return 0;
00049    }
00050 #endif
00051 
00052    /****************************************/
00053    /****************************************/
00054 
00055    CCI_GripperActuator::CCI_GripperActuator() :
00056       m_fLockState(0.0f) {}
00057 
00058    /****************************************/
00059    /****************************************/
00060 
00061    void CCI_GripperActuator::SetLockState(Real f_lock_state) {
00062       UNIT.TruncValue(f_lock_state);
00063       m_fLockState = f_lock_state;
00064    }
00065 
00066    /****************************************/
00067    /****************************************/
00068 
00069    void CCI_GripperActuator::Lock() {
00070      SetLockState(1.0f);
00071    }
00072 
00073    /****************************************/
00074    /****************************************/
00075 
00076    void CCI_GripperActuator::Unlock() {
00077      SetLockState(0.0f);
00078    }
00079 
00080    /****************************************/
00081    /****************************************/
00082 
00083 #ifdef ARGOS_WITH_LUA
00084    void CCI_GripperActuator::CreateLuaState(lua_State* pt_lua_state) {
00085       CLuaUtility::StartTable(pt_lua_state, "gripper");
00086       CLuaUtility::AddToTable(pt_lua_state, "_instance", this);
00087       CLuaUtility::AddToTable(pt_lua_state, "lock", &LuaGripperLock);
00088       CLuaUtility::AddToTable(pt_lua_state, "unlock", &LuaGripperUnlock);
00089       CLuaUtility::EndTable(pt_lua_state);
00090    }
00091 #endif
00092 
00093    /****************************************/
00094    /****************************************/
00095 
00096 }
```

---

Generated on 10 Jul 2018 for ARGoS by 
 1.6.1
